# Relationship between Habitual Caffeine Consumption, Attentional Performance, and Individual Alpha Frequency during Total Sleep Deprivation

**DOI:** 10.3390/ijerph20064971

**Published:** 2023-03-11

**Authors:** Michael Quiquempoix, Catherine Drogou, Mégane Erblang, Pascal Van Beers, Mathias Guillard, Pierre-Emmanuel Tardo-Dino, Arnaud Rabat, Damien Léger, Mounir Chennaoui, Danielle Gomez-Merino, Fabien Sauvet

**Affiliations:** 1 Institut de Recherche Biomédicale des Armées (IRBA), 91223 Brétigny sur Orge, Francefabien.sauvet@gmail.com (F.S.); 2 URP 7330 VIFASOM, Université Paris Cité, Hôtel-Dieu, 75004 Paris, France; 3 Laboratoire de Biologie de l’Exercice pour la Performance et la Santé (UMR LBEPS), Université d’Evry, 91025 Evry-Courcouronnes, France; 4 APHP, Hôtel-Dieu, Centre du Sommeil et de la Vigilance, 75004 Paris, France

**Keywords:** sleep deprivation, vulnerability, PVT performance, EEG alpha activity, caffeine consumption

## Abstract

(1) Background: Caffeine is a psychostimulant that is well known to mitigate the deleterious effects of sleep debt. Our aim was to assess the effects of acute caffeine intake on cognitive vulnerability and brain activity during total sleep deprivation (TSD), taking into account habitual caffeine consumption. (2) Methods: Thirty-seven subjects were evaluated in a double-blind, crossover, total sleep deprivation protocol with caffeine or placebo treatment. Vigilant attention was evaluated every six hours during TSD using the psychomotor vigilance test (PVT) with EEG recordings. The influence of habitual caffeine consumption was analyzed by categorizing subjects into low, moderate, and high consumers. (3) Results: The PVT reaction time (RT) increased during TSD and was lower in the caffeine condition vs. the placebo condition. The RT was shorter in the low-caffeine consumers compared to moderate and high consumers, regardless of conditions and treatments. The TSD-related increase in EEG power was attenuated by acute caffeine intake independently of habitual caffeine consumption, and the individual alpha frequency (IAF) was lower in the high-consumption group. The IAF was negatively correlated with daytime sleepiness. Moreover, a correlation analysis showed that the higher the daily caffeine consumption, the higher the RT and the lower the IAF. (4) Conclusions: A high level of habitual caffeine consumption decreases attentional performance and alpha frequencies, decreasing tolerance to sleep deprivation.

## 1. Introduction

Several professions are exposed to prolonged wakefulness, resulting in an increased risk of accident that is linked to increased daytime sleepiness and decreased cognitive performance [1,2,3]. In experimental protocols on total sleep deprivation, vigilant attention, assessed by the psychomotor vigilance task (PVT), is the most affected cognitive capability [4,5]. Changes in the individual EEG alpha and theta power after prolonged wakefulness were also found to be related to subjective sleepiness and PVT performance [6,7,8].

Caffeine is used to counteract sleep-loss-related neurobehavioral impairment and has been shown to increase alertness and decrease daytime sleepiness [9]. Caffeine is a non-selective antagonist of adenosine receptors (mainly A1 and A2A) which modulates glutamatergic, cholinergic, dopaminergic, serotoninergic, and noradrenergic neurotransmission [10]. In laboratory studies on sleep deprivation, acute caffeine administration helped to restore cognitive function, often by preventing a decrease in sustained attention, with doses ranging from 200 to 600 mg [7,8,11,12,13]. The acute intake of caffeine reduced sleepiness and EEG theta activity during wakefulness [6,7,14]. Under non-deprived sleep conditions, the acute intake of 200 mg of caffeine caused a significant reduction in total EEG power at the frontal, central, and parieto-occipital electrode positions of both hemispheres when the subjects kept their eyes open, and the absolute power of the slow and fast alpha and slow beta activities was diminished in various regions of the brain [15]. Subsequently, the acute intake of 250 mg of caffeine was associated with a global reduction in the eyes-closed resting-state EEG power in the alpha band and a global increase in alpha frequency [16]. In most of the above-mentioned studies, authors included non- or low-caffeine consumers or subjects following a caffeine withdrawal prior to experimental protocols [17].

However, while the effects of acute caffeine intake are widely described, the effects of habitual high daytime consumption are not well documented and understood. In this way, the categorization of participants depending on their habits of caffeine consumption has been suggested to be essential when analyzing the physiological effects (e.g., sympathetic nerve activity and blood pressure) of caffeine intake [18]. Interestingly, the impact of acute caffeine consumption on decision making and risk taking has been demonstrated to be dependent on several factors, including habitual caffeine use (categorized as low, moderate, and high caffeine consumption) [19]. In a recent study, Weibel et al. (2021) demonstrated that regular caffeine intake during the daytime (450 mg per day) delays REM sleep promotion in healthy subjects, evidenced by prolonged REM sleep latency and delayed REM sleep accumulation [20]. The same research group also demonstrated that pure attentional processing can be enhanced by acute caffeine consumption but not by daily caffeine consumption, with the exception of increasing the associated neural activity [21]. To our knowledge, no studies have analyzed the effects of acute caffeine intake on PVT performance or a topographic quantitative EEG during total sleep deprivation (TSD) as a function of habitual caffeine consumption.

The aim of our study was to evaluate the influence of habitual caffeine consumption on PVT performance during total sleep deprivation, using an EEG spectral analysis during PVT at its circadian nadir of performance impairment in a crossover condition of acute caffeine or placebo intake.

## 2. Materials and Methods

### 2.1. Participants

Thirty-seven subjects, aged between 18 and 55 years, were included in this study. The subjects did not have medical, psychiatric, or sleep disorders. Other exclusion criteria included physical, sleep, or mental health troubles based on (I) the hospital anxiety and depression scale (HAD)(≥11) [22], (II) a significant medical history, (III) the Epworth sleepiness scale (ESS) (>10) [23], (IV) the Pittsburg sleep quality index (PSQI) (>8) [24], (V) the morningness–eveningness questionnaire (<31 or >69) [25], and (VI) the habitual time in bed per night (<6 h). We purposely chose to exclude participants with an extreme chronotype because the study presented here was part of a multi-objective project with a main objective of analyzing the influence of 14 single-nucleotide polymorphisms (SNPs), including those of the circadian clock gene PERIOD3 (PER3), on cognitive responses to sleep deprivation [8]. The subjects were asked to not use medications with sleep-related side effects, illicit drugs, or abuse alcohol. Subjects did not travel between time zones within 7 days and did not work in shifts in the 2 weeks prior to the study. Subjects were required to complete a sleep–wake schedule during the week preceding the study and were asked to maintain their habitual caffeine consumption until they entered the laboratory protocol.

The subjects’ habitual caffeine consumption was assessed using a questionnaire [26], which included the following beverages and caffeine-containing foods: coffee with caffeine, tea, cola, other carbonated beverages with caffeine, and chocolate. For each item, participants were asked to indicate how often, on average, they had consumed a given amount of each food or drink in the past year [19]. Participants could choose from nine frequency categories (never, 1–3 per month, 1 per week, 2–4 per week, 5–6 per week, 1 per day, 2–3 per day, 4–5 per day, and 6 or more per day). Typical doses in milligrams (Mayo Clinic- http://www.mayoclinic.com/health/caffeine/AN01211, accessed on 4 March 2018) were assigned to each, and an approximate daily intake was obtained.

### 2.2. Study Design and Testing Conditions

This study was conducted in the sleep laboratory of the Armed Forces Biomedical Research Institute (IRBA), Brétigny sur Orge, France. The ambient temperature was controlled and maintained at 22 ± 1 °C during all experiments. The brightness of the lighting was maintained between 150 and 200 lux during the awake periods, and the lights were turned off during sleep periods. Meals and caloric intake were standardized for all subjects (2600 kcal/day).

**Figure 1:**
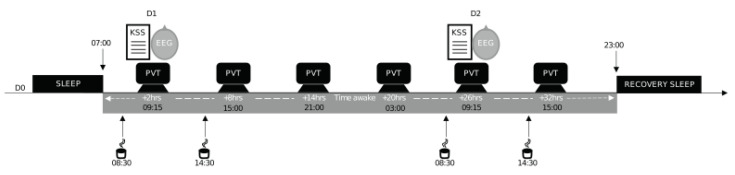
Study design. Experimental design including a habituation and training day (D0), a baseline day (D1), and a total sleep deprivation (TSD) day beginning on D1 at 23:00 until D2 at 21:00. Subjects completed the psychomotor vigilance task (PVT) every 6 h and were EEG-recorded during the PVT at 09:15 at D1 and D2. They also completed the Karolinska sleepiness scale (KSS). (↓) 08:30 and 14:30; acute placebo or caffeine (2.5 mg/kg) intake.

The experimental, in-laboratory protocol included (I) a habituation/training day (D0), (II) a baseline day (D1) beginning at 07:00, and (III) a total sleep deprivation (TSD) day beginning on D1 at 23:00 and lasting until 21:00 at D2, followed by an overnight recovery period so that the subjects could safely leave the laboratory site (Figure 1). Subjects were welcomed in groups of 4 at approximately 16:00 on D0. During tests, subjects were always under the visual surveillance of research staff members.

When participants were not engaged in testing, meals, or sleep periods, they were not allowed to exercise or use tobacco, alcohol, or other psychoactive substances. However, they were allowed to read, watch videos, speak with other participants or staff members, and play games, following a pre-established program. In addition, we used a wrist actigraph to check that the subjects stayed awake during the 38 h of continuous wakefulness. Subjects were asked to maintain their habitual consumption of caffeine for two weeks prior each laboratory period.

### 2.3. Acute Administration of Caffeine

This study was double-blind, crossover, and placebo-controlled for the administration of caffeine (two conditions: caffeine (CAF) and placebo (PBO)) with a 2-week washout period between the two conditions during which subjects returned to their off-protocol lifestyle. Either caffeine or a placebo was administered in a decaffeinated beverage twice per day (at 08:30 and 14:30, corresponding to 1.5 and 7.5 h of prolonged wakefulness, respectively) on D1 and D2 (Figure 1).

For the caffeine condition, each participant received 2.5 mg per kg body weight of caffeine powder mixed in a decaffeinated beverage at 08:30 and 14:30 (5 mg/kg/day). The caffeine powder was pre-measured by the project supervisor. This amount of caffeine powder was chosen for its attention-enhancing properties in sleep-deprived conditions (2.5–8 mg/kg of caffeine) [27]. Tests were performed 45 min after caffeine ingestion.

### 2.4. Daytime Sleepiness and Sustained Attention Measurements

Subjects completed the psychomotor vigilance task (PVT) every 6 h from 09:15 on D1 to 15:00 on D2. The KSS was filled in, and the EEG was recorded at 09:15 only on D1 and D2, corresponding to 2 and 26 h of prolonged wakefulness, respectively (Figure 1). All subjects had a systematic habituation period for tests at D0 (habituation/training day) in order to reduce a learning bias during the first set of tests.

We used a computer-based version of the 10 min PVT [28]. Subjects were instructed to respond by clicking the left mouse button as soon as the visual stimulus appeared (incremental millisecond counter), without making false starts. The inter-stimulus interval was randomized between 2 and 10 s. The reaction time (RT) was quantified in milliseconds for a 1 s period, and the response was regarded as valid if the RT was ≥100 ms.

The Karolinska sleepiness scale (KSS) is a subjective scale used to grade a subject’s sleepiness from 1 to 9, with 1 indicating “extremely alert” and 9 indicating “extremely sleepy” [29]. The computer version used in this study allowed the subject to choose from nine options.

### 2.5. EEG Recording and Analysis

The EEG was recorded during the 10 min PVT to evaluate the theta, alpha, and beta power at 2 h (D1, 09:15) and at 26 h (D2, 09:15) of prolonged wakefulness.

The EEG was recorded at 19 scalp sites, according to the international 10–20 system (Fp1, Fp2, F7, F3, Fz, F4, F8, T7, C3, Cz, C4, T8, P7, P3, Pz, P4, P8, O1, and O2), using a Siesta 802 (Compumedics Limited, Victoria, Australia). The EEG was recorded continuously at a sampling rate of 512 Hz and was referenced with bridged mastoidal electrodes. The data were re-referenced during preprocessing with a common average. The electrodes were interfaced with the scalp using EC2 gel (Grass Technologies, AstroNova, Inc., West Warwick, RI, USA), and impedances were kept below 10 kOhm during the entire session. The EEG was installed approximately 20–30 min before the first cognitive task (PVT) and was supplemented with EC2 gel if needed to prevent dry electrodes during the recordings.

The EEG data were analyzed in Matlab (Mathworks, Natick, MA, USA), using the Fieldtrip toolbox [30] and custom codes. The data were band-stop filtered between 48 and 52 Hz to remove electrical noise. The data were then high-pass filtered above 0.1 Hz and locally detrended. Blink artefacts were removed by computing an independent component analysis (ICA, Fieldtrip), and movement artefacts were removed by visual inspection. Bad electrodes were systematically rejected; if more than 3 (out of 19) electrodes were removed, the subject was excluded from EEG analysis. The EEG parameters of 3 out of 37 subjects were not analyzed (*n* = 34 subjects for EEG analysis). The EEG theta (4–8 Hz), alpha (8–12 Hz), and beta (12–20 Hz) power were assessed using the continuous Morlet wavelets transform. Regions of interest (ROIs) represent the means of the grand averaged (all subjects) power over the frontal (Fp1, Fp2, F7, F3, Fz, F4, F8), centro-temporal (T7, C3, Cz, C4, T8), and parieto-occipital (P7, P3, Pz, P4, P8, O1, O2) regions. The alpha frequency (AF) was assessed by computing Equation (1) at each electrode site:

AF_electrode_ = (∑ (pow(fq) × fq))/(∑ (pow(fq)) (1)

where fq represents the frequency (8–12 Hz) and pow(fq) represents the power at the corresponding frequency. The individual alpha frequency (IAF) was then obtained by averaging the AFs over all (*n* = 19) electrodes (2) [31]:

IAF = (∑electrode (AF_electrode_))/(n_electrodes_) (2)

### 2.6. Statistical Analysis

The statistical analyses were computed using Jamovi (version 1.6.15, 2022). Values were expressed as the mean ± SEM. Values for the PVT RT, KSS, IAF, and EEG band power were analyzed using a linear mixed model including fixed effects for awakening duration (repeated measures ranging from 2 to 32 h), acute treatment (caffeine or placebo, repeated measure), and habitual caffeine consumption (non-repeated measure).

Secondly, post hoc analyses were performed to compare the differences between the caffeine consumption groups. Differences in the subjects’ characteristics between the groups of habitual caffeine consumption were evaluated using a one-way ANOVA (3 levels). The significance level was set at *p* < 0.05. As we observed a significant difference between groups for age, the effect of groups on cognitive performance was assessed using a mixed linear model including caffeine consumption groups, awakening (repeated measure), and a random effect on age.

Repeated measures correlations were computed between KSS, daily caffeine consumption levels, RT, and IAF. All post hoc analyses were performed using Student’s *t*-test on the categories of low (0–50 mg/day), moderate (51–300 mg/day), and high (>300 mg/day) caffeine consumption. The post-hoc analyses were corrected with a Holm–Bonferroni procedure and are noted as pholm.

## 3. Results

Thirty-seven subjects (15 women, 40.5 %) were analyzed. Three of the subjects were excluded from the EEG analysis due to a low EEG signal quality. The subjects were 30.2 ± 4.4 years old, with an average weight of 66.9 ± 4.1 kg, a mean habitual caffeine consumption of 212 ± 66 mg/day, a mean physical activity of 3.0 ± 0.5 h/week, and a habitual sleep duration of 7.16 ± 0.5 h per night. Seven (18.9%) of our subjects were habitual consumers of tobacco. We first analyzed cognitive performance in relation to daily caffeine consumption and then second in relation to categorized caffeine consumption. The latter was performed on the basis of previous criteria [19], and participants were categorized as follows: 10 were low-caffeine consumers (0–50 mg per day), 11 were moderate (51–300 mg per day), and 16 were high (>300 mg per day).

### 3.1. First Analysis of Cognitive Performance in Relation to the Daily Caffeine Consumption

**Figure 2:**
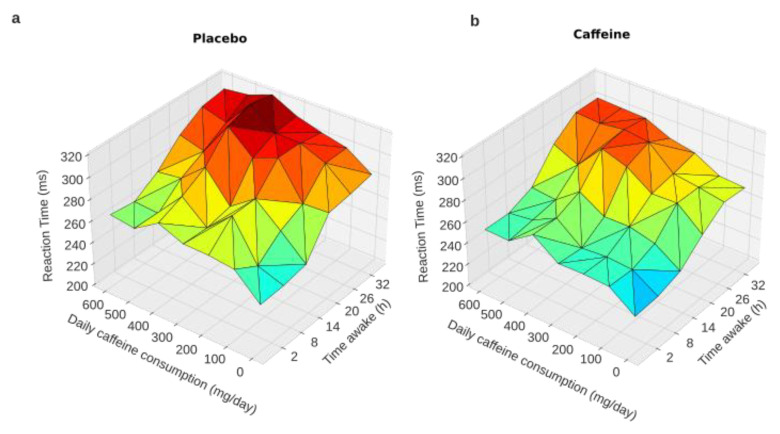
PVT reaction times during total sleep deprivation as a function of caffeine daily consumption. Graphical representation of the linear mixed-model analysis showing a trend of an interaction between habitual daily caffeine consumption and time awake (from 2 to 32 h) in the conditions of acute placebo (**a**) and caffeine intake (**b**).

Reaction time (RT) during PVT. A linear mixed-model analysis with habitual daily caffeine consumption as a continuous variable was used. The model demonstrated significant main effects for acute treatment (CAF or PBO conditions) (F = 26.72, *p* = 0.001), awakening (F = 184.62, *p* = 0.001), and habitual daily caffeine consumption (F = 5.125, *p* = 0.03). No statistical interactions were observed for acute treatment × awakening, awakening × daily caffeine consumption, acute treatment × daily caffeine consumption, or the triple interaction of awakening × acute treatment × daily caffeine consumption (Table 1). This is illustrated in Figure 2, which shows that the higher the habitual daily caffeine consumption, the higher the RT, either in the conditions of an acute placebo (Figure 2a) or caffeine intake (Figure 2b). 

### 3.2. Secondary Analysis of Cognitive Performance Plus EEG Characteristics in Relation to Habitual Caffeine Consumption Groups

#### 3.2.1. Subjects’ Characteristics According to Habitual Caffeine Consumption

The age means between the groups were statistically different (F = 3.4, *p* = 0.04, Supplementary Table S1), with post-hoc tests revealing that the high consumers were significantly older than the moderate consumers (Games-Howell, *p* = 0.03) and not low consumers (Games-Howell, *p* = 0.252). The statistical analysis found no differences between groups for sex (Chi^2^, X^2^ = 0.11, *p* = 0.8), physical activity (ANOVA, F = 0.43, *p* = 0.51), body weight (ANOVA, F = 1.7, *p* = 0.2) or habitual sleep time (F = 0.51, *p* = 0.61) (Table S1).

#### 3.2.2. Reaction Time (RT) during PVT

**Figure 3:**
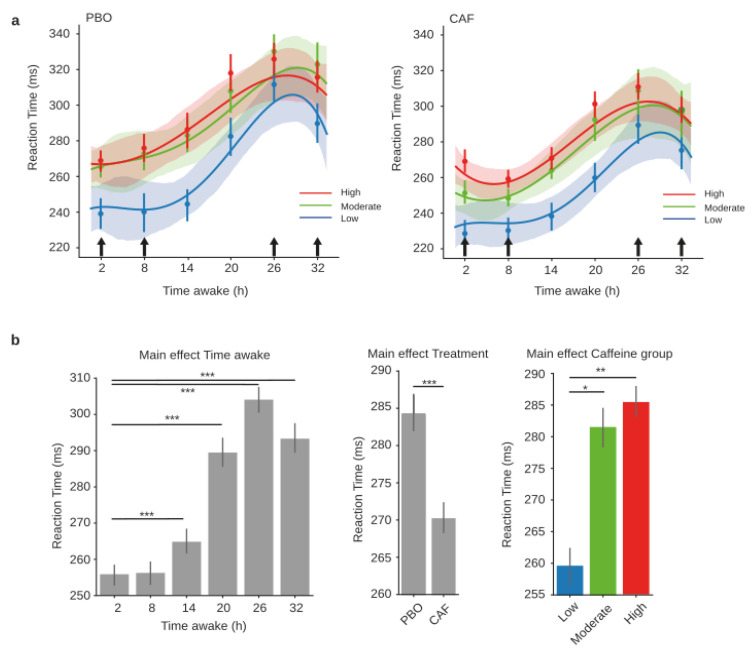
PVT reaction times during total sleep deprivation for the three caffeine consumption groups. Polynomial curve fitting of mean reaction times (RT, mean ± S.E.M) as a function of time spent awake (h) for low (blue), moderate (green), and high (red) habitual caffeine consumption groups in the placebo (**a**, left panel) or caffeine (**a**, right panel) conditions. (**b**) Main effects of time awake, treatment, and caffeine group, respectively, on reaction times obtained from the mixed-model analysis. Black arrows represent acute caffeine or placebo intake. * *p* < 0.05, ** *p* < 0.01, *** *p* < 0.001.

According to the three groups of habitual caffeine consumers (low, moderate, and high), the RT analysis showed significant main effects for treatment (CAF or PBO conditions) (F = 15.52, *p* = 0.001), day (F = 47.88, *p* < 0.001), and caffeine group (F = 5.34, *p* = 0.009), and demonstrated no statistical interactions for treatment x day (F = 1.70, *p* = 0.134), treatment x caffeine group (F = 0.64, *p* = 0.529), day × caffeine group (F = 1.27, *p* = 0.265), or the triple interaction of treatment × day × caffeine group (F = 0.8, *p* = 0.63) (Figure 3a, PBO on the left and CAF on the right). Post-hoc comparisons for the main effects revealed a significantly higher RT from 14 h to 32 h compared to 2 h of continuous awake time ( t = −3.45, *p* = 0.001; t = −9.69, pholm < 0.001; t = −13.14, pholm < 0.001; and t = −10.21, pholm < 0.001, respectively) (Figure 3b, left panel). The comparisons also showed a lower RT in the acute CAF treatment condition compared to PBO (t = −5.12, pholm < 0.001) (Figure 3b, middle panel), and a lower RT for the low-consumption habitual caffeine consumers compared to the moderate (t = −2.35, pholm = 0.015) and high consumers (t = −3.01, pholm = 0.049) (Figure 3b, right panel).

#### 3.2.3. EEG Power Spectra

**Figure 4:**
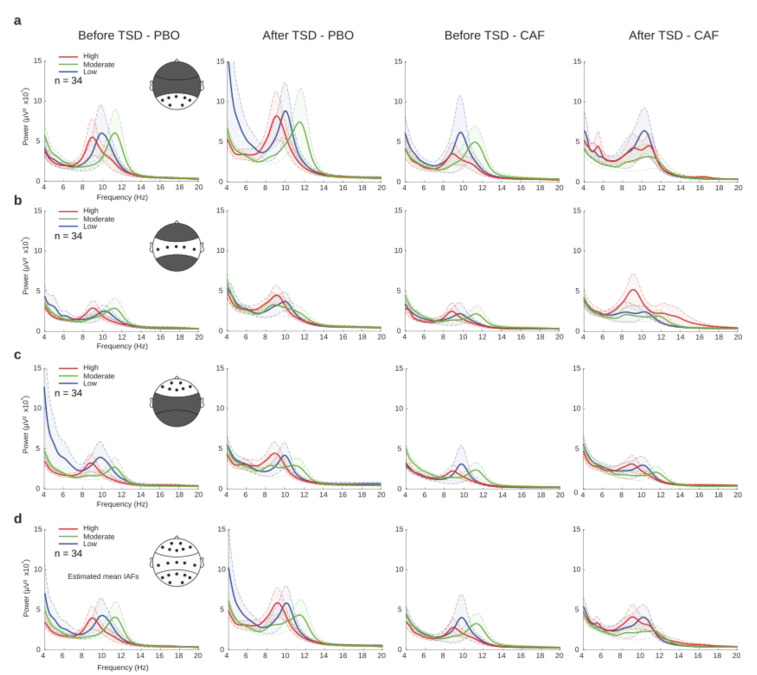
EEG power spectra before (D1 day) and after (D2 day) total sleep deprivation (TSD) for placebo (PBO) and caffeine (CAF) conditions at different regions of interest (ROIs). Power spectra (µV^2^) of each group of habitual caffeine consumption (low (blue), moderate (green), and high (red)) in parieto-occipital (**a**), centro-temporal (**b**), or frontal (**c**) regions, and the global field potential (**d**).

In order to identify EEG correlates during PVT before and after TSD for the PBO and CAF conditions, respectively, power spectral analyses were performed and averaged across the regions of interest (ROIs): parieto-occipital, centro-temporal, and frontal (Figure 4a–c). The theta, alpha and beta mean powers (µV^2^) were statistically compared using a linear mixed-model analysis for the three main effects (treatment, day, and caffeine group) and their interactions. Significant effects of day were observed within all three ROIs for theta and alpha, and only in the parieto-occipital and frontal regions for beta (Table 2). Main treatment effects were found for alpha in all three ROIs, for theta in the centro-temporal region, and in the frontal region for the beta power. Finally, we noticed significant treatment × day interactions for theta in the parieto-occipital and centro-temporal regions and for alpha in the parieto-occipital region. A visual inspection of the global power spectra revealed a trend of different individual alpha frequencies (IAFs) between the daily caffeine consumption groups (Figure 4d). This parameter was further explored.

#### 3.2.4. Relationships between Subjective Sleepiness, Caffeine Daily Consumption, PVT Performances, and Alpha Frequencies

**Figure 5:**
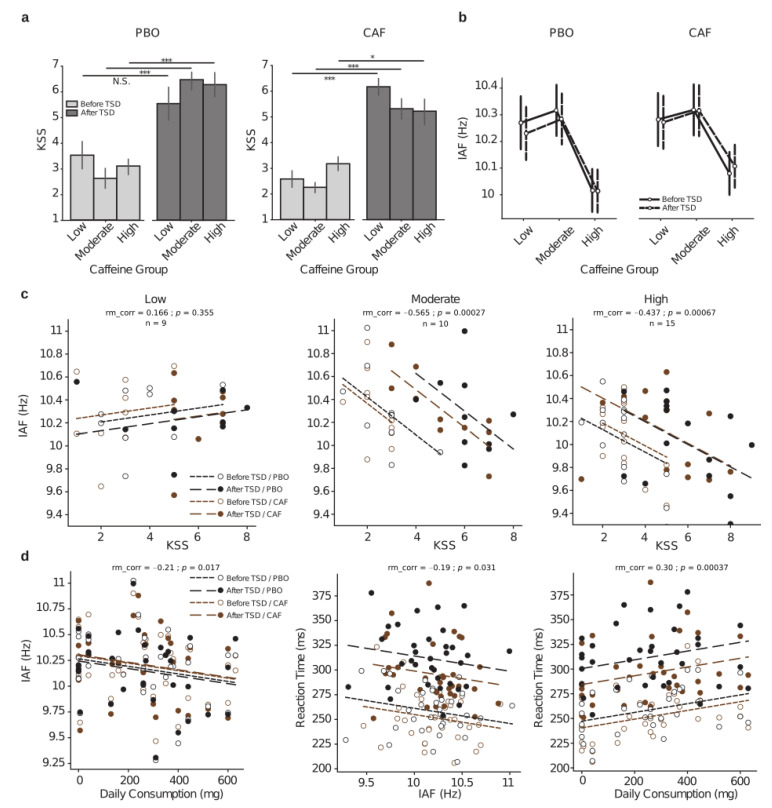
Relationships between subjective sleepiness (KSS) scores, PVT reaction times, and individual alpha frequencies as function of the habitual caffeine consumption in response to total sleep deprivation (TSD). (**a**) KSS scores before (D1 day, light gray) and after (D2 day, dark gray) TSD either in the placebo (PBO, left panel) or caffeine (CAF, right panel) condition for the three groups of habitual caffeine consumption. (**b**) Individual alpha frequency (IAF in Hz) before (D1 day, in solid black lines) and after (D2 day, in dashed black lines) TSD in the two treatment conditions (PBO, left panel; CAF, right panel) for the three groups of habitual caffeine consumption. (**c**) Repeated measures correlations between IAF and KSS score for all Day × Treatment conditions (black dots and lines for placebo (PBO), brown dots and lines for caffeine (CAF), empty dots and densely dashed lines for before TSD, and solid dots and loosely dashed lines for after TSD) for the three respective groups of habitual caffeine consumption (low at left, moderate at middle, and high at right); rm_corr represents the common within-session association. (**d**) Repeated measures correlations between IAF and habitual daily consumption levels (left), reaction time and IAF (middle) and reaction time and daily consumption levels (right); dots and dashed lines legends are the same as in (**c**). * *p* < 0.05, *** *p* < 0.001.

The Karolinska sleepiness scale (KSS) was used to assess subjective sleepiness at 2 h and 26 h of continuous wakefulness (i.e., before and after TSD). We observed significant main interactions on treatment (F = 5.22, *p* = 0.026) and day (F = 110.35, *p* < 0.001) but not for caffeine groups (F = 0.36, *p* = 0.69), and a significant triple interaction (F = 4.33, *p* = 0.017). More specifically, the KSS scores were significantly increased after TSD relative to before TSD for all groups in the PBO or CAF conditions except for the group of low-caffeine consumers in PBO (PBO: t = 2.98, pholm = 0.28, t = 6.002, pholm < 0.001, t = 5.73, pholm < 0.001; CAF: t = 5.39, pholm < 0.001, t = 4.79, pholm < 0.001, t = 3.67, pholm = 0.042 for low, moderate, and high consumers, respectively) (Figure 5a). We did not find any statistical differences based on the caffeine group or treatment in KSS scores (Table S2). As previously mentioned, we further explored the impact of habitual caffeine consumption on the individual alpha frequency (IAF). We performed an analysis of covariance using age as a covariate and found a significant main effect of the caffeine group (F = 6.79, *p* = 0.002), demonstrating a significantly higher IAF for low and moderate consumers when compared to high consumers (t = 2.84, pholm = 0.01, t = 3.45, pholm= 0.002, respectively). No statistical differences were found between the low and moderate groups (t = −0.6, pholm = 0.55) (Figure 5b). We found no differences between treatments (F = 0.49, *p* = 0.48), days (F = 0.033, *p* = 0.86), treatment × day (F = 0.004, *p* = 0.83), treatment × caffeine group (F = 0.01, *p* = 0.88), day × caffeine group (F = 0.004, *p* = 0.95), or their triple interaction (F = 0.002, *p* = 0.97).

Although there were no day or treatment effects on the IAF, significant negative correlations were observed between the IAF and KSS scores within the moderate- and high-caffeine consumer groups (Figure 5c, middle and right, respectively).

We then explored whether linear correlations may occur between cognitive performances (RT-PVT), IAF, and daily caffeine consumption at the individual level (Figure 5d). We first corroborated our above results by identifying a significant negative correlation between IAF and the individuals’ habitual daily caffeine consumption (Figure 5d left), with higher caffeine consumption associated with a lower IAF. Finally, there were significant negative correlations between the RT and IAF and between the RT and the individuals’ daily consumption (Figure 5d, middle and right, respectively).

## 4. Discussion

The present study examined the relationship between habitual caffeine consumption and reaction time (i.e., vigilant attention) and its neurophysiological correlates during total sleep deprivation (TSD) by performing the psychomotor vigilance task (PVT) at six times during TSD and recording the EEG during the PVT task at the nadir of performance impairment, both with and without acute caffeine intake.

First, we showed that although the acute beneficial effect of caffeine on the reaction time during TSD does not depend on habitual caffeine consumption, low-caffeine consumers are faster than moderate- and high-caffeine consumers, reflecting less impairment to their attentional processes, including during total sleep deprivation. This was shown with subjects categorized into three groups of caffeine consumption (0–50 mg/day; 51–300 mg/day; and >300 mg/day), as defined in our previous study [26] and after an age-corrected statistical analysis as the high-caffeine consumers were significantly older. Second, we demonstrated that the reaction time has a negative linear correlation with the EEG individual alpha frequency (IAF) (e.g., the lower the reaction time is, the stronger the IAF is), and a strong positive correlation with the individuals’ daily caffeine consumption. The latter results are corroborated by a strong negative correlation between the IAF and the individuals’ daily caffeine consumption. The correlation analysis between the habitual caffeine consumption and reaction time or the IAF EEG-specific parameter was performed independent of the caffeine groups in order to eliminate any possible arbitrariness related to categorization.

Most studies on the effects of caffeine on cognitive performance under circumstances of sleep-deprivation-related impairment were performed with acute caffeine intake [11,12]. In these studies, the subjects were low to moderate caffeine consumers (not exceeding 400 mg per day) and were asked to abstain from caffeine and other psychoactive substances for at least 12 h before each test session. In our experiment, we purposely asked our subjects to maintain their habitual caffeine consumption until they entered the laboratory protocol in order to remain as close as possible to the lifestyle habits of the general population. This was done in order to evaluate the effects of caffeine on vigilance under conditions of sleep deprivation and to be able to address future advice to certain professional populations (e.g., physicians and surgical staff, military, etc.). To our knowledge, this is the first study to demonstrate that vigilant attention during TSD is impaired as soon as the habitual caffeine consumption exceeds 50 mg per day, even if an acute intake of 2.5 mg/kg twice per day (corresponding to 350 mg per day for a 70 kg person) is beneficial. Indeed, the statistical analysis evidenced a main treatment (caffeine/placebo) effect without significant interactions with habitual caffeine consumption, suggesting similar responses to acute caffeine intake regardless of daily habit. Regarding a possible interaction with a caffeine withdrawal effect for subjects in the placebo condition, one would expect moderate and high caffeine users to be more degraded than low users during prolonged wakefulness. In our study, the short duration of the placebo condition did not imply caffeine withdrawal as several studies agree that withdrawal is achieved after several days [11,12,32]. Our results thus confirmed the acute beneficial effect of caffeine during TSD as reported in numerous studies, including ours [8,11,12]. Nonetheless, there was a significant main effect of habitual caffeine consumption demonstrated, with low consumers having shorter reaction times than moderate and high consumers. This leads us to conclude that a high habitual consumption of caffeine is associated with lower vigilant attention before and during sleep deprivation. A few alerts have been issued on the deleterious effects of chronic caffeine consumption for adults, particularly on physiological effects such as blood pressure [18]. The deleterious effect of habitual caffeine consumption compared to acute intake has been described for risk-taking behavior in children and adolescents, populations who should be protected from this type of behavior [19]. These results demonstrate that the acute intake of 2 mg/kg if caffeine decreases risk-taking behavior in moderate-caffeine consumers while increasing it in high-caffeine consumers.

The electrophysiological correlates of acute caffeine intake have also been widely described with a consensus on its global decreasing effects on EEG powers during cognitive tasks in sleep-deprived and non-sleep-deprived subjects [6,7,8] or in resting states with the eyes closed [16]. In agreement with the literature, we found significant main treatment (caffeine/placebo) effects on theta, alpha, and beta power bands which were reflected by decreasing powers with acute caffeine intake. We found no significant effects on EEG power bands for the habitual caffeine group nor interactions with the acute treatment (and/or day or sleep deprivation effect). Nevertheless, the visual inspection of the power spectra (Figure 3) revealed an interesting trend of slower alpha frequencies in high consumers compared to the two other groups. This can be analyzed through the individual alpha frequency (in Hz) calculation (and/or the corresponding frequency of alpha peak amplitude, the individual alpha peak frequency). This characteristic of the alpha rhythm is well known to correlate with different aspects of cognition, nociception, or age [33,34]. It is also related to mental fatigue [35,36,37] and is not sensitive to acute caffeine intake [38]. In addition, the power density in the 6.25 to 9.0 Hz band of the waking EEG recorded at the same four time points during a 40-h period of sustained wakefulness showed a significant positive correlation with the fatigue ratings [37]. Our objective was thus to explore the relationships between IAF, habitual caffeine consumption, and attentional performances (RT-PVT) before and during total sleep deprivation. We first assessed the link between the IAF and the KSS (Karolinska sleepiness score), which is used to rate the perception of fatigue under condition of degraded sleep, such as in operational aircraft pilots [39,40]. Åkerstedt’s team demonstrated that median reaction time, alpha and theta power density, and the alpha attenuation coefficients were increased with an increase in the KSS score [41]. As expected, our results showed significant increases in the KSS score after TSD [37] without an acute caffeine effect or differences between the caffeine groups. On the contrary, IAF was significantly lower in high-caffeine consumers than in the low and moderate groups, and no difference was found between the low and moderate consumers. The latter result may be explained by the categorization of the groups, with possible overlap between them. In addition to the small sample size, this overlap may have artificially masked the differences between low and moderate consumers. Interestingly, we found neither a main effect of treatment (caffeine/placebo) nor an effect of sleep deprivation on IAF, which is in agreement with Tiffin et al. (2006) for the lack of an acute effect of caffeine, whereas we lacked data analysis of IAF after TSD.

The separate analyses of repeated-measure linear correlations between the IAF and KSS scores for each caffeine group showed strong and significant negative correlations for moderate and high consumers and no correlations for low consumers. Considering the increase in IAF, which may reflect a higher mental effort, we hypothesize that moderate and high consumers failed to maintain their IAF with increasing psychophysiological constraints (e.g., mental fatigue reflected by their increasing subjective sleepiness during TSD), and that they performed worse as a result. As previous studies reported that the average IAPF (peak frequency) predicted global cognition [42] and that the slower alpha oscillatory cycling explains global cognitive deficits in schizophrenia [43], we performed a correlation analysis between the IAF and PVT reaction times, adding an analysis with daily caffeine consumption at the individual level. We showed (i) a strong negative relationship between IAF and the individuals’ daily consumption levels, (ii) a negative relationship between reaction times and IAF, and, finally, (iii) a positive relationship between reaction times and the individuals’ daily consumption levels. Our results not only confirmed that the higher IAF, the better the reaction time, but also that a higher caffeine consumption is linked to a lower IAF and a worse reaction time. The significant linear relationship between the IAF and the individuals’ daily consumption confirmed our above hypothesis on the absence of differences between low and moderate consumers. Together, these data suggest that a high habitual consumption of caffeine may decrease individuals’ ability to increase their alpha frequency depending on the task demand and context, thereby decreasing their reaction time with respect to vigilant attention.

Several mechanisms may be implicated in our observed results concerning the effects of habitual caffeine consumption on PVT reaction time in sleep-deprived subjects with or without acute caffeine administration, i.e., a higher reaction time and a lower task-related EEG individual alpha band frequency (IAF) when habitual caffeine consumption exceeds 50 mg per day (corresponding to less than one expresso). The action of caffeine, an adenosine receptor antagonist, mainly affects the quality of sleep and the cardiovascular system [44]. Extracellular adenosine concentrations increase during prolonged periods of wakefulness and lead to increased sleep pressure. The response of high caffeine users to a caffeine challenge is likely to be quite different due to the fact that chronic caffeine use impacts the adenosine receptor system. Several studies on receptor binding have shown either an upregulation of A1 or A2A receptors or an increase in the affinity of either system, suggesting that adaptations to the adenosine receptor system do occur with regular caffeine consumption [45]. For Jacobson et al. (1996), cognitive responses differed after acute vs. chronic exposure to either selective agonists or antagonists of A1 receptors, and chronic administration of a selective A1 receptor antagonist even slightly impaired memory acquisition in mice. With respect to the relationship between caffeine consumption and sleep, it has been evidenced that regular caffeine consumption in adolescents may lead to reduced sleep depth, measured by EEG slow-wave activity, and reduced alpha activity without changes occurring in sleep architecture (i.e., sleep stages) and continuity [46]. Thus, although the total sleep time was no different between the three groups of caffeine consumers in our study, their deep sleep may have been reduced, which may lead to alterations in the recovery processes that are favorable to vigilant attention.

However, our study also had several limitations that must be addressed in future studies. Our results showed a relationship between habitual caffeine consumption and vigilant attention in healthy, sleep-deprived subjects. Higher executive functions, such as inhibition or working memory, should be further explored in this context. Other EEG markers related to task performance (e.g., evoked potential, induced time–frequency maps, etc.) might reveal more subtle links between chronic caffeine consumption and cognitive functioning. Moreover, we cannot ignore a possible impact of caffeine withdrawal on our results (i.e., participants arrived at the laboratory the day before the experiment), as high caffeine consumers potentially faced withdrawal in the placebo condition.

## 5. Conclusions

In conclusion, habitual caffeine consumption affects attentional performance and individual alpha frequency in healthy subjects before and during total sleep deprivation. Future studies should assess higher executive processes in the same type of experimental protocol. Our results demonstrate an interest in systematically assessing daily caffeine consumption habits and control-confounding effects on the performance of vigilance-related tasks. We also suggest that our data be considered in the military operational context to individualize advice in terms of caffeine consumption and sleep debt management.

## Figures and Tables

**Table 1:** Effects of habitual caffeine consumption, awakening, and treatment on PVT performances. A linear mixed-model analysis was performed on PVT reaction times (RT). Significant *p* values are flagged in bold.

*p*	Den df	Num df	F	
0.03	35	1	5.125	Caffeine Consumption
0.001	35	1	26.72	Treatment
0.001	35	1	184.62	Awakening
0.066	331	1	3.407	Treatment × Awakening
0.59	35	1	0.288	Caffeine Consumption × Treatment
0.291	35	1	1.147	Caffeine Consumption × Awakening
0.72	331	1	0.127	Caffeine Consumption × Treatment × Awakening

**Table 2:** Effects of treatment condition (PBO vs. CAF), day (D1 vs. D2), and caffeine consumption groups (low, moderate, and high) on EEG parameters. A linear mixed-model analysis was performed for the EEG theta, alpha, and beta power at each region of interest (ROI), assessing fixed effects of treatment, day, and caffeine group as well as their respective interactions. Significant *p* values are flagged in bold.

Frontal			Centro-Temporal			Parieto-Occipital				
*p*	df	F	*p*	df	F	*p*	df	F		
0.150	1.0	2.23	**0.030**	1.0	5.42	0.090	1.0	2.99	Treatment	Theta Power
**0.002**	1.0	10.10	**<0.001**	1.0	14.76	**0.029**	1.0	5.14	Day	
0.726	2.0	0.32	0.980	2.0	0.02	0.338	2.0	1.12	Caffeine Group	
0.858	1.0	0.03	**0.009**	1.0	6.90	**0.010**	1.0	6.57	Treatment × Day	
0.268	2.0	1.37	0.645	2.0	0.44	0.569	2.0	0.57	Treatment × Caffeine Group	
0.579	2.0	0.55	0.491	2.0	0.73	0.491	2.0	0.73	Day × Caffeine Group	
0.117	2.0	2.15	0.435	2.0	0.83	0.053	2.0	2.94	Treatment × Day × Caffeine Group	
**0.006**	1.0	8.55	**0.042**	1.0	4.49	**0.049**	1.0	4.18	Treatment	Alpha Power
**0.003**	1.0	8.94	**<0.001**	1.0	15.68	**0.003**	1.0	9.48	Day	
0.997	2.0	0.00	0.811	2.0	0.21	0.996	2.0	0.00	Caffeine Group	
0.273	1.0	1.20	0.178	1.0	1.82	**0.019**	1.0	5.46	Treatment × Day	
0.462	2.0	0.79	0.597	2.0	0.53	0.529	2.0	0.65	Treatment × Caffeine Group	
0.545	2.0	0.61	0.513	2.0	0.68	0.900	2.0	0.11	Day × Caffeine Group	
0.186	2.0	1.68	0.136	2.0	1.99	0.552	2.0	0.59	Treatment × Day × Caffeine Group	
**0.017**	1.0	6.33	0.640	1.0	0.22	0.143	1.0	2.26	Treatment	Beta Power
**<0.001**	1.0	16.02	0.075	1.0	3.35	**0.009**	1.0	7.34	Day	
0.793	2.0	0.23	0.293	2.0	1.28	0.928	2.0	0.07	Caffeine Group	
0.951	1.0	0.00	0.746	1.0	0.11	0.068	1.0	3.45	Treatment × Day	
0.329	2.0	1.15	0.794	2.0	0.23	0.661	2.0	0.42	Treatment × Caffeine Group	
0.526	2.0	0.65	0.435	2.0	0.85	0.343	2.0	1.09	Day × Caffeine Group	
0.323	2.0	1.15	0.074	2.0	2.60	0.480	2.0	0.74	Treatment × Day × Caffeine Group	

## Data Availability

Data can be obtained by request of the corresponding author.

## Supplementary Materials

The following supporting information can be downloaded at: https://www.mdpi.com/article/10.3390/ijerph20064971/s1, Table S1: Subjects’ characteristics; Table S2: Post-hoc comparisons of treatment (PBO vs CAF) x day (D1 vs D2) x caffeine group (Low, Moderate, High) on KSS scores.
